# Thrust-efficiency limits of a swimming tail with variable chordwise flexural rigidity

**DOI:** 10.1038/s41598-025-88365-x

**Published:** 2025-02-06

**Authors:** Hossam Alqaleiby, Muhammad R. Hajj

**Affiliations:** Davidson Laboratory, Department of Civil, Environmental and Ocean Engineering, Stevens Institute of Technology, Hoboken, NJ 07030 USA

**Keywords:** Fish locomotion, Chordwise stiffness, Finite-element method, Stiffness profile, 3D unsteady vortex lattice method, Aerospace engineering, Mechanical engineering

## Abstract

Emulating oscillations performed by natural swimmers can provide different functionalities than those of propeller-based underwater robots. Yet, to successfully accomplish specific missions under limited power, there is a need to design efficient bio-inspired robots. Adding an appropriate level of flexibility to flapping caudal fins (tails) of robots emulating the thunniform swimming mode has been shown to enhance the thrust generation over a finite range of the flapping frequency. Still, in many cases, adding flexibility to increase thrust generation may require increased input power, which may cause a significant reduction in the efficiency. These observations lead to the concept of enhanced performance by varying the stiffness of the tail as in the case of natural swimmers. This study is concerned with assessing the impact of varying the chordwise stiffness on the tail deflection and flow dynamics, including contributions of added mass and circulation forces to thrust generation and their impact on efficiency. The simulation data are used to identify specific flow dynamics and tail deflections associated with the enhanced thrust generation and/or efficiency, and to define a performance limit expressed as the maximum efficiency as a function of the thrust coefficient.

## Introduction

By mimicking oscillations and undulations performed by natural swimmers, swimming robots can achieve improved performance and functionalities that are different from those of propeller-based underwater robots. One swimming mode capable of maintaining high cruising speeds for long periods is the thunniform mode^1–5^ where fish, such as tuna, generate thrust by oscillating their tail^6,7^. In this mode, the lateral oscillations of the relatively stiff caudal fin produce more than 90% of the thrust^7^. Another oscillatory swimming mode is the ostraciiform mode where all thrust is generated by periodic oscillations of the caudal fin^7^. For oscillatory bioinspired swimmers, the thrust generated by the oscillating tails and their efficiency depend on the shape, stiffness, aspect ratio of the tail, as well as the frequency and amplitude of the force or torque applied at the root or leading edge of the tail. Although the development and design of a bioinspired robot depend mainly on its mission needs, optimizing its functionalities remains essential. Given the differing performance metrics such as thrust generation and efficiency and their dependence on many variables as noted above, this optimization must be based on analysis that takes into consideration these variables and their effects on the metrics. For instance, thrust generated by periodic oscillations of rigid tails can be estimated using Theodorsen’s analysis of pitching and/or heaving airfoils^8^, which yields contributions of circulatory and added mass components to the thrust based on potential flow theory. Garrick^9^ used Theodorsen theory to derive analytical expressions for the thrust, power, and efficiency of such air or hydrofoils. Flow characteristics associated with thrust generation have been addressed in experimental and numerical studies. Koochesfahani^10^ related thrust generation to a reverse Von Karman wake and showed a linear dependence between pitch frequency and axial flow of the wake. Anderson et al.^11^ determined that the maximum thrust is obtained when the Strouhal number, based on the displacement (peak to peak) of the trailing edge, forward speed, and forcing frequency, lies between 0.25 and 0.4, which is the range of many natural swimmers.

Because the caudal fins of many fish passively change shape during swimming, there has been significant interest in determining the effects of added flexibility on thrust generation and its efficiency. In seminal works based on potential flow theory, Lighthill^12,13^ and Wu^14,15^ explained the dynamics of the swimming of fish and propulsive mechanisms. In particular, Wu^15^ demonstrated analytically that flexible hydrofoils can be more efficient than rigid ones in producing thrust. The results of many numerical^16–18^ and experimental studies^19,20^ have confirmed that an appropriate level of uniform flexibility of flapping tails can improve thrust generation and its efficiency for different geometries and aspect ratios. Based on experiments conducted in a water channel, Dewey et al.^19^ determined that the improvement in the thrust of the flexible tails depends on the effective stiffness defined as the ratio of elastic and fluid forces. The numerical simulations of Hussein et al.^18^ showed that, over a specific range of excitation frequencies, the trailing edge of a flexible tail undergoes peak-to-peak amplitudes higher than those of its rigid counterpart, which enhances thrust generation. However, this ability is limited to a specific range of tail flexibility below which thrust generation is reduced. Their results also showed that, depending on flexibility, increasing thrust may require larger power, leading to reduced efficiency. Regarding flow quantities, their results showed that although flexibility impacts the generated circulatory force, its effects on the added mass component are more pronounced, signifying its role in enhancing thrust generation.

Observing that fish tails have variable stiffness, several studies have considered the effects of varying the stiffness of the tail in the chordwise direction on fish swimming^21–24^. In general, it has been found that gradually varying the stiffness from the leading edge to the tip improves the propulsion performance in comparison to the performance of tails with uniform stiffness distribution^25–28^. Kancharala and Philen^26^ conducted experimental and theoretical studies to seek the optimal chordwise stiffness profiles of self-propelled fins undergoing pitching and heaving motions. They observed that varying-stiffness fins generate larger thrust and efficiency than uniform-stiffness fins because of their larger curvatures and trailing edge deflections. Reddy et al.^25^ modeled a flexible trapezoidal caudal fin inspired by the bluegill sunfish as rigid segments connected by torsion springs along its length to study the effect of stiffness distribution on propulsion performance. They calculated the hydrodynamic force on each fin segment by summing the added mass and drag force components using the blade element method. Their results showed that thrust can be enhanced by tuning the chordwise stiffness.

The objectives of this study are to (1) assess the relation between the generated force and required power, in terms of thrust and efficiency, and the beam deflections, in terms of peak-to-peak amplitude and trailing edge slope, of uniform and non-uniform stiffness tails, (2) investigate the effects of varying tail stiffness over a broad range of leading edge and distributed stiffness values on its deformation characteristics, (3) determine contributions by the flow quantities, circulatory and added mass force components, as affected by the tail deformation on the relation between thrust generation and efficiency, and (4) derive an expression for the performance limit in terms of maximum efficiency for a set thrust value that can be generated by tuning the stiffness of a flexible tail in the chordwise direction. Toward these objectives, we perform numerical simulations and analyze the performance of a flexible tail subjected to pitching excitation at its root while moving forward at a constant swimming speed. The simulations cover a broad range of varying stiffness configurations. The analysis, which addresses relations between thrust generation, efficiency, tail deformation, and flow quantities, provides an approach to evaluate and improve the initial configuration design of swimming robots expected to emulate the thunniform mode.

## Methods

**Fig. 1:**
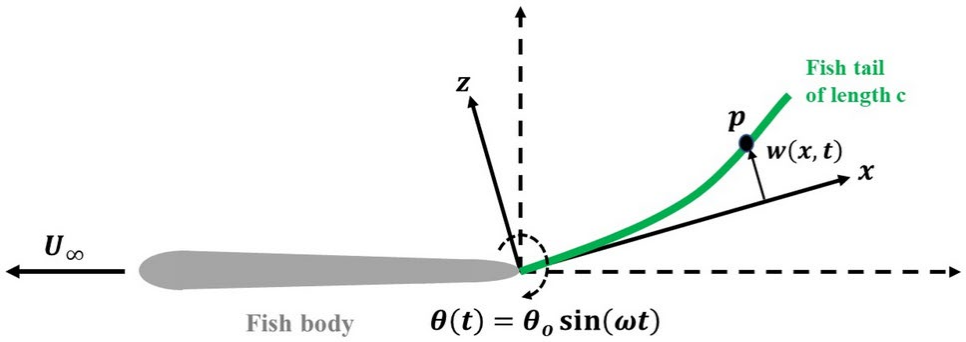
Schematic representation of a bioinspired swimmer propelled by pitching tail oscillations.

Two approaches have mostly been followed to model variable stiffness of flapping tails in the chordwise directions. These include a tail segmented by one or more linear torsional springs^29–31^ or a flexible beam modeled as a linear elastic beam^18,32–34^. One advantage of modeling the tail as an elastic beam is that it offers a framework for considering intrinsic flexibility and shape simultaneously^35^. Different-fidelity flow models have been used to calculate the hydrodynamic forces originating from flapping a flexible tail. These include linear unsteady airfoil theory^36^, vortex shedding models^18,29,31,37^, or Navier-Stokes/Boltzman solvers^38,39^. To simulate the fluid-structure interaction, it is required to capture the tail within the fluid domain^40^. Here, we solve the coupled equations of the tail deformation and hydrodynamic loads using an implicit strong coupling approach^18^. The rectangular tail (chord *c* x span *d*) is moving with a uniform forward speed $U_\infty$ and is excited at its root by sinusoidal pitching, having an amplitude $\theta _o$ and a frequency $\omega$, and represented by $\theta (t)=\theta _o sin(\omega t)$ as shown in Fig. 1. The pitching amplitude varies between $-8^{\circ }$ and $8^{\circ }$.

For the range of flexibility levels considered in this work and based on the experimental results of Dewey et al.^41^, we neglect the flexibility in the spanwise direction. As such, we use the Euler-Bernoulli beam theory to derive the governing equation for the elastic deflection of the tail due to the pitching excitation at its root under hydrodynamic loading. Following the derivation of Hussein et al.^18^, we write the governing equation as follows:

1 $$\begin{aligned} \frac{\partial ^2}{\partial x^2}\left( EI(x) \frac{\partial ^2 w}{\partial x^2}\right) - \frac{\partial }{\partial x}\left( T(x) \frac{\partial w}{\partial x}\right) + m\left( \ddot{w}-\ddot{\theta } x - \dot{\theta }^2 w \right) = F_H (w,\dot{w},\ddot{w}) \end{aligned}$$

where the deflection is represented by *w*(*x*, *t*), $\theta$ is the amplitude of the excitation applied at the root, *EI*(*x*) is the chordwise varying flexural rigidity of the tail, $F_H$ is the local hydrodynamic force per unit length and *T*(*x*) is the tension force in the tail that has a maximum value at the connecting point or the root and zero value at the free trailing edge, and is given by

2 $$\begin{aligned} \begin{aligned} T(x)&= \frac{m \dot{\theta }^2}{2} \left( c^2 - x^2\right) - m \int _{x}^c \left( \ddot{\theta }w + 2\dot{\theta }\dot{w}\right) dx \approx \frac{m \dot{\theta }^2}{2} \left( c^2 - x^2\right) \end{aligned} \end{aligned}$$

To discern the effects of the different terms, we define the non-dimensional quantities

3 $$\begin{aligned} \begin{aligned}&x^{*} = \frac{x}{c},&\quad w^{*} = \frac{w}{c},&\quad AR=\frac{d}{c}&\text { and}&\quad t^{*} = \frac{tU_{\infty }}{c} \end{aligned} \end{aligned}$$

Substituting these quantities into equation (1) yields the nondimensional form of the governing equation of motion as:

4 $$\begin{aligned} \begin{aligned}&\Pi _1 AR \frac{\partial ^4 w^{*}}{\partial x^{{*}^4}} + \mu ^{-1} AR \frac{\partial }{\partial x^{*}} \biggl (\frac{1}{2}\left( \frac{d \theta }{dt^{*}}\right) ^2 \left( 1-x^{{*}^2}\right) \frac{\partial w^{*}}{\partial x^{*}}\biggr ) + \\&+ \mu ^{-1} AR \left( \frac{\partial ^2 w^{*}}{\partial t^{{*}^2}} - \frac{d^2 \theta }{dt^{{*}^2}} x^{*} - \left( \frac{d\theta }{dt^{*}} \right) ^2 w^{*} \right) = F_H^* (w^{*}, \dot{w}^{*}, \ddot{w}^{*}, k, St) \end{aligned} \end{aligned}$$

where

5 $$\begin{aligned} \begin{aligned}&\Pi _1 = \frac{Eh^3}{12 \rho _f U_{\infty }^2 c^3},&\quad \mu = \frac{\rho _f c}{\rho _s h},&\quad St = \frac{f A}{U_\infty } = \frac{\omega A}{2 \pi U_\infty },&\quad k = \frac{\omega c}{2 U_\infty }, \text { and}&\quad F^{*}_H = \frac{F_H}{\rho _f U^2_{\infty } c}\\ \end{aligned} \end{aligned}$$

Here $\Pi _1$ is the effective stiffness (inverse of flexibility) and is defined as the ratio of the elastic restoring forces to the fluid pressure forces and $\mu$ is the mass ratio defined as the ratio of the mass of moving solid body and that of the fluid. Given that the unsteady hydrodynamic force will depend on the tail length *c*, swimming speed $U_\infty$, and excitation frequency $\omega$, we express the non-dimensional hydrodynamic force $F^{*}_H$ as a function of the Strouhal number *St*, which describes the tail kinematics by relating excitation frequency, the peak-to-peak amplitude and forward speed, and of the reduced frequency *k*, which is the nondimensional excitation frequency. In the following analysis, we consider swimming performance over a broad range of leading edge stiffness between $3\times 10^{-3} Nm^2$ and $25\times 10^{-3} Nm^2$, corresponding to nondimensional effective stiffness $\Pi _1$ values between 0.44 and 3.67, and reduced frequencies up to 5 while keeping the mass ratio constant at 81, which is representative of natural swimmers.

We use the three-dimensional unsteady vortex lattice method (3D UVLM) approach^42,43^ that captures the distribution of added mass forces in the chordwise direction, the distribution of bound circulation, wake circulation, and induced drag that are associated with the swimming action. The total force is determined by summing the pressure force at all panels, where the pressure is calculated using the unsteady Bernoulli equation. Details about implementing the three-dimensional UVLM, the boundary conditions, Kutta condition, and pressure calculations can be found in Hussein et al.^18^ and Alqaleiby et al.^44^. The power required to flap the tail, referred to as the input hydrodynamic power and denoted as $P_{in}$, is calculated as the sum of the dot product of the local torque and angular velocity vectors at each panel. The output power, i.e. the propulsive power noted as $P_{out}$, is calculated as the dot product of the total force and forward velocity vectors. These quantities are then used to calculate the thrust coefficient $C_T$, power coefficient $C_P$, and the propulsive efficiency $\eta _p$ according to:

6 $$\begin{aligned} C_T = \frac{\overline{P}_{out}}{\frac{1}{2} \rho U_\infty ^3 d c} = \frac{\overline{F}_X U_{\infty }}{\frac{1}{2} \rho U_\infty ^3 d c}, \qquad C_P = \frac{\overline{P}_{in}}{\frac{1}{2} \rho U_\infty ^3 d c} = \frac{\overline{\sum _{i}^{N_x} \sum _{j}^{N_y} \left( \vec {{\textbf {r}}}_{oi, j} \times \vec {{\textbf {F}}}_{i,j}\right) \cdot \vec {\varvec{\Omega }}}}{\frac{1}{2} \rho U_\infty ^3 d c}, \text { and} \qquad \eta _p = \frac{\overline{P}_{out}}{\overline{P}_{in}}, \end{aligned}$$

where $\overline{()}$ refers to mean value over several flapping cycles, $N_x$ and $N_y$ are respectively the number of panels in the chordwise and spanwise directions, $\vec {{\textbf {r}}}_{oi, j}$ is the position vector from the leading edge to the center of each panel, $\vec {{\textbf {F}}}_{i, j}$, is the force vector from each panel, and $\vec {\varvec{\Omega }}$ is the angular velocity vector.

The finite element method is used to discretize the space operator in the equation of motion (1) by multiplying the residual by a weight function ($\tilde{w}$) and integrating over the generic finite element length. After performing these steps and rearranging the terms, the equation is written as

7 $$\begin{aligned} \begin{aligned}&\int _{0}^{l}\frac{\partial ^2}{\partial x^2}\left( EI(x) \frac{\partial ^2 w}{\partial x^2}\right) \tilde{w}^{T} dx - \int _{0}^{l} \frac{\partial }{\partial x}\left( T(x) \frac{\partial w}{\partial x}\right) \tilde{w}^{T} dx + m \int _{0}^{l} \left( \ddot{w} - \dot{\theta }^2 w\right) \tilde{w}^{T} dx \\ &= \int _{0}^{l} \left( F_H \tilde{w}^{T} + m\ddot{\theta } x \tilde{w}^{T} \right) dx \end{aligned} \end{aligned}$$

which is rewritten in the form^18^

8 $$\begin{aligned} {[} {\textbf {M}}] \{\ddot{{\textbf {q}}}\} + [{\textbf {K}}] \{{\textbf {q}}\} = \{{\textbf {F}}({\textbf {q}}, \dot{{\textbf {q}}}, \ddot{{\textbf {q}}})\} \end{aligned}$$

where $[{\textbf {M}}]$ is the mass matrix, $[{\textbf {K}}]$ is the stiffness matrix, and $\{{\textbf {F}}({\textbf {q}},\dot{{\textbf {q}}},\ddot{{\textbf {q}}})\}$ is the time-dependent forcing vector. Equation (8) is then directly integrated in time using the Newmark method, which is implemented by defining a system of equations for ${\textbf {q}}$, and $\ddot{{\textbf {q}}}$^18,45,46^. We then follow the strong coupling approach, which requires rewriting the system of equations as algebraic equations and use the Newton-Raphson method to solve these equations based on a displacement convergence criterion, as detailed by Hussein et al.^18^ and Alqaleiby et al.^44^. The above model and solution follow the procedure developed by Hussein et al.^18^ that was validated against the experimental observations by Dewey et al.^19^.

## Results

### Effects of stiffness distribution on tail performance

**Fig. 2:**
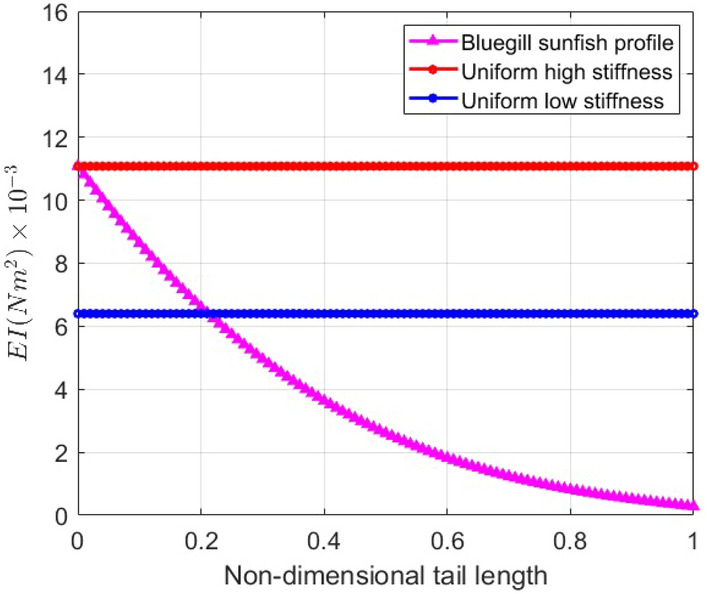
Flexural rigidity distribution of the considered nonuniform stiffness (NUS), uniform high stiffness (UHS) and uniform low stiffness (ULS) flexible tails.

As a first step in the evaluation of the effects of varying the chordwise stiffness or rigidity on the performance metrics and associated flow and beam deflection characteristics, we consider a square tail that has varying flexural rigidity, referred to as the nonuniform stiffness (NUS) tail. At the root, *EI* is set to $11\times 10^{-3} N m^2$ ($\Pi _1 = 1.58$). This value drops to $0.28\times 10^{-3} N m^2$ ($\Pi _1 = 0.041$) at the trailing edge as shown in Fig. 2. The nonuniform stiffness distribution is adopted from the measurements of Tangorra et al.^22^ for bluegill sunfish fin ray no. 4, scaled to the tail length considered here, and fitted to a cubic polynomial profile as

9 $$\begin{aligned} \begin{aligned}&EI(x) = -9.7984 x^3 + 2.842 x^2 - 0.2964 x + 0.01108, \ \ \ \ \ \ \ \ \ \ \ \ \ \ \ \ \ \ \ \ 0< x< c, \hspace{0.5 cm} \text {or}\\&EI(x^{*}) = -0.0071 x^{*3} + 0.023 x^{*2} - 0.0267 x^{*} + 0.01108 \ \ \ \ \ \ \ \ \ \ \ \ \ \ \ \ \ \ \ \ 0< x^{*} < 1 \end{aligned} \end{aligned}$$

where the chord length *c* is equal to 9 cm. To complete the assessment, we will compare the performance metrics, beam deflection, and flow quantities of the NUS tail to those of two tails having uniform rigidity of $6.4\times 10^{-3} N m^2$ ($\Pi _1 = 0.92$) and $11\times 10^{-3} N m^2$ ($\Pi _1 = 1.58$) and are respectively referred to as the uniform low stiffness (ULS) and uniform high stiffness (UHS) tails. We also compare the performance of the three tails with that of a rigid tail.

**Fig. 3:**
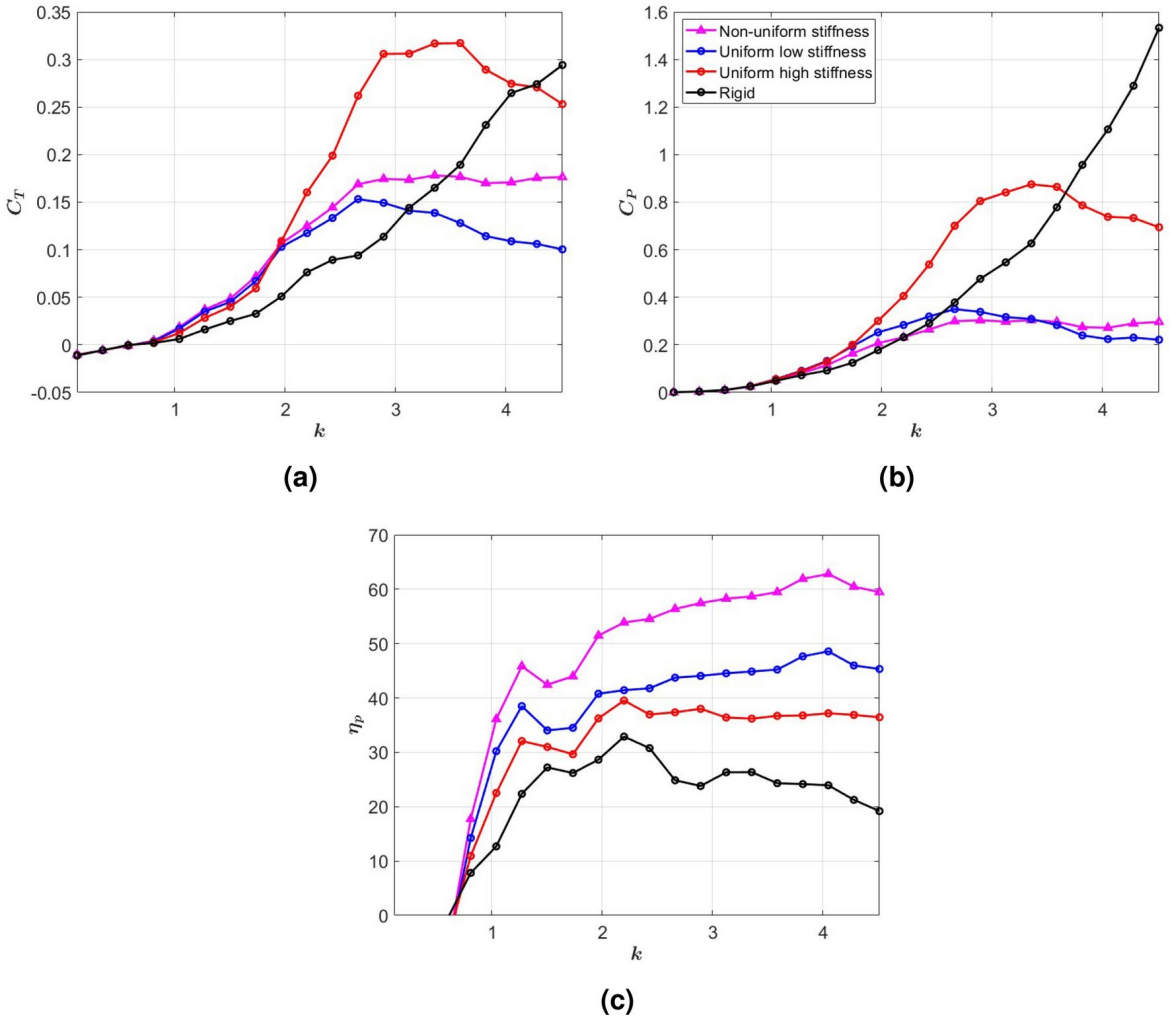
(**a**) Thrust coefficient $C_T$, (**b**) power coefficient $C_P$, and (**c**) propulsive efficiency $\eta _p$ of the NUS, UHS, ULS and rigid tails versus reduced frequency.

We plot in Fig. 3 the thrust and hydrodynamic power coefficients, and efficiency as defined in equation (6) of the four tails over the range of reduced frequencies $k < 4.5$. The plots in Fig. 3a show no appreciable thrust generation in the low reduced frequency range $k < 1$ by any of the four tails. Although thrust generated by the fully rigid tail increases as the reduced frequency is increased from $k = 1$ to $k = 4.5$, the more significant increase in the required power shown in Fig. 3b makes it as the least efficient tail (Fig. 3c). The ULS and UHS tails, respectively, exhibit a greater thrust between $k = 1$ to $k = 3$ and between $k = 1$ and $k = 4$ compared to the rigid tail. However, this thrust drops rapidly after peaking at a reduced frequency near $k = 2.8$ for the ULS tail and $k=3.6$ for the UHS tail. Although the thrust generated by the UHS tail is significantly greater (double) than that generated by the ULS tail over a specific range of excitation frequencies (Fig. 3a), it requires almost three times the hydrodynamic power required by the ULS tail (Fig. 3b), making the UHS less efficient than the ULS tail, as shown in Fig. 3c. Clearly, a uniform stiffness setting that enhances thrust by a flexible tail is not the same setting that would enhance efficiency, which limits the capability of uniform stiffness tails. Comparing the thrust generated by the NUS and ULS tails, we observe that it is the same in the low range of reduced frequencies $k < 2.8$. Beyond this value, the thrust produced by the ULS decreases as the reduced frequency is increased. In contrast, the thrust generated by the NUS tail remains relatively large (almost constant) in the reduced frequency range $k > 2.8$. Considering the almost equal required hydrodynamic power by these two tails as shown in Fig. 3b, it is concluded that the NUS tail can generate greater thrust than the ULS tail without a need for increased power, i.e. with a higher efficiency than the ULS tail. This significant improvement in its efficiency is actually realized over the whole range of reduced frequencies above $k = 1$, as shown in Fig. 3c.

**Fig. 4:**
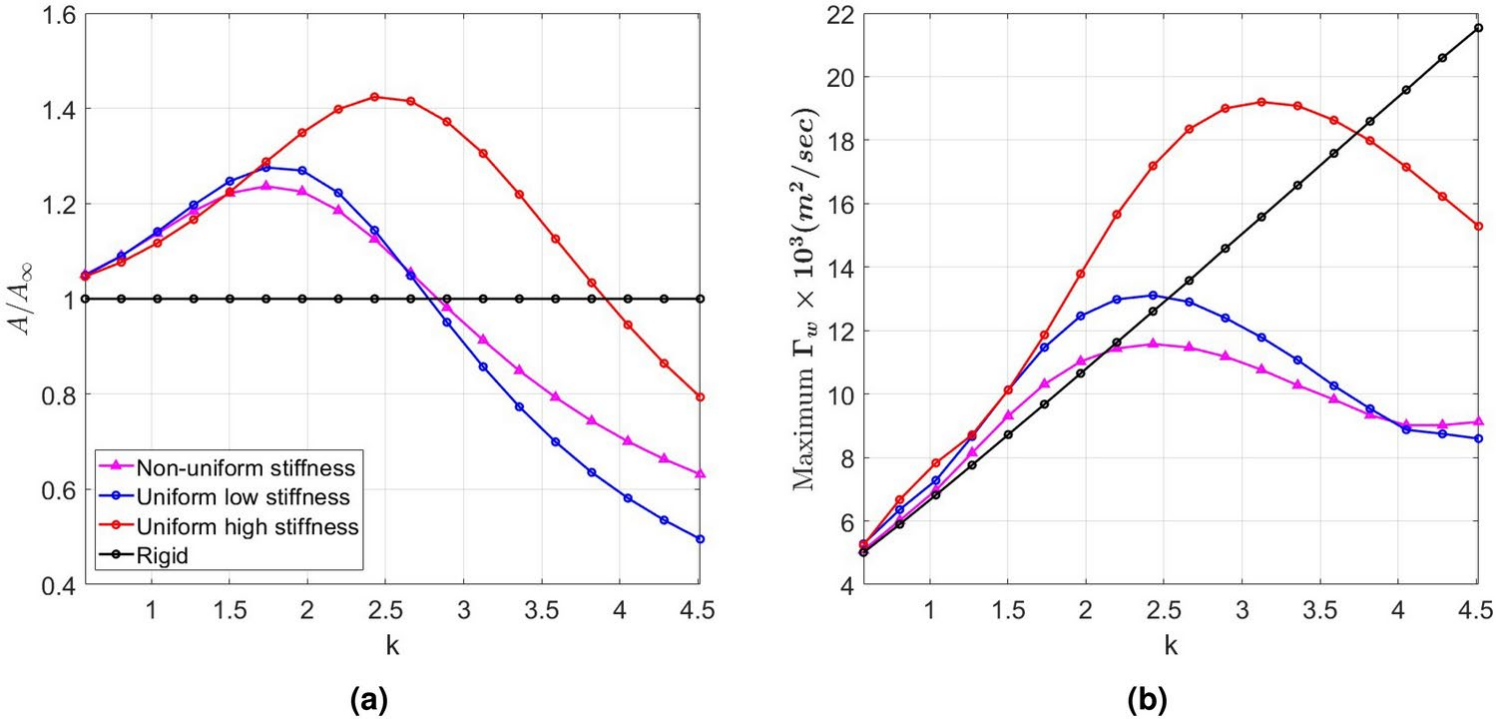
(**a**) Peak-to-peak amplitude, and (**b**) maximum wake circulation versus reduced frequency, *k*.

Next, we investigate the reasons for this improved performance by the NUS tail by associating it with specific aspects of the tail deformation and flow quantities. One measure of tail deformation is the distance traveled by the trailing edge during half of the oscillation period, which is referred to as the peak-to-peak amplitude. Figure 4a shows this amplitude for the three flexible tails normalized with the peak-to-peak amplitude of the rigid tail. Comparing the graphs in Figs. 3a and 4a, it is observed that the higher thrust generated by the UHS tail in the range of reduced frequencies $k < 4$ and the ULS tail over the range of reduced frequencies $k < 3$ matches their ability to produce larger peak-to-peak amplitudes than the rigid tail in these regions. In both cases, once their peak-to-peak amplitudes drop to values below that of the rigid tail, their thrust coefficients drop to values below that of the rigid tail. It is important to note here that the maximum peak-to-peak amplitude in each case, which is assumed to occur when the excitation is near the corresponding natural coupled frequency, does not correspond with the maximum generated thrust, indicating that peak thrust is not associated with resonance conditions but rather with improved hydrodynamic performance or favorable tail deflection. As for the NUS tail, although its peak-to-peak amplitude variation with the reduced frequency is very close to that of the ULS tail, its thrust generation is larger than that of the ULS tail even when the peak-to-peak amplitude is reduced below that of the rigid tail. Furthermore, this improved thrust generation is associated with a smaller required power (Fig. 3b) and, thus, a higher efficiency (Fig. 3c).

Given that thrust is associated with the tail’s wake, we plot in Fig. 4b the generated maximum circulation in the wake for the four tails. By comparing these graphs with the graphs in Fig. 3a, it is observed that, over the range of frequencies where higher thrust is generated, the ULS and UHS stiffness tails generate higher levels of circulation in the wake when compared to the circulation generated by the rigid tail. Also, comparing the plots in Fig. 4b with those in Figs. 3b and 4a, we note that the increased circulation of the wake by the rigid or uniform stiffness tails is directly related to the increased peak-to-peak amplitudes and the power requirement. In contrast, the NUS tail, which generates lower wake circulation levels than the ULS tail, yields a larger thrust than that tail at lower peak-to-peak amplitude and required power. This indicates that varying the chordwise stiffness can support a larger thrust without the need to increase the wake circulation, which is directly related to the increased power requirement to flap the tail.

**Fig. 5:**
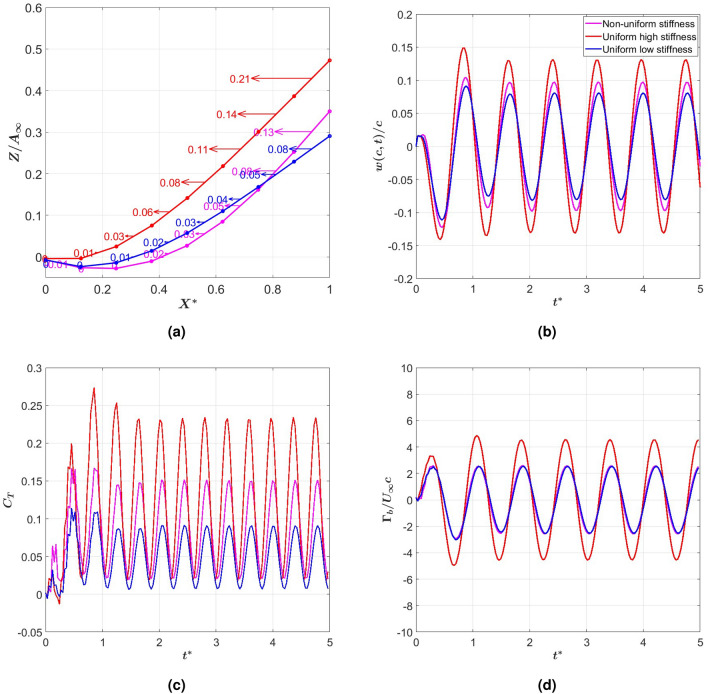
(**a**) Distribution of the thrust coefficient along the elements of the NUS, ULS and UHS tails at their maximum deformation position, and time histories of the (**b**) tail’s tip deflection, (**c**) thrust generated by last tail element, and (**d**) bound circulation at last element of the tail at $k = 4$.

To determine the deflection characteristics of the NUS tail that generate enhanced thrust, we show in Fig. 5a the distribution of the thrust coefficient along the elements of the tail when it experiences maximum deformation at the reduced frequency of $k = 4$. The plots show that, in all cases, pressure forces in the thrust (or swimming) direction increase from the root to reach a maximum near the trailing edge. Comparison of the plots shows that the UHS tail exhibits a larger peak-to-peak amplitude than the ULS tail. In contrast, the NUS tail exhibits a deflection with a higher slope than those of the ULS and UHS tails at the trailing edge, which results in a larger component of the pressure force in the swimming direction while also increasing the peak-to-peak amplitude when compared to the ULS tail. The impact of this favorable deflection on thrust generation is demonstrated in the time series of the tip deflection in Fig. 5b and thrust coefficient in Fig. 5c. When comparing the plots of the three figures, it is noted that the larger tip deflection supports a larger thrust generation by the last element of the tail near the trailing edge. However, comparing the relative increase in thrust and peak-to-peak amplitude, it is noted that the higher slope (0.68) exhibited by the NUS tail compared to the slope of the ULS tail (0.44) is a more effective approach to increase thrust because it favorably redirects the pressure force more toward thrust generation. In Fig. 5d, we compare the time histories of bound circulation at the last element of the three tails. The plot shows that the UHS tail has the highest circulation amplitude, which can be related to the larger thrust but also the larger power requirement. On the other hand, both the NUS and ULS tails have the same circulation magnitude, which shows that the enhanced thrust generation by the favorable deflection or curvature of the NUS tail is not due to enhanced circulation, which requires more power, but rather due to enhanced added mass effects resulting from a more favorable beam deflection.

### Performance limit

The above results demonstrated that thrust generation can be enhanced by distributing the stiffness, which yields a more favorable beam deflection and peak-to-peak amplitude for enhanced efficiency with higher contribution to thrust from the added mass. Still, developing an initial design based on expected capabilities requires assessment of performance metrics over a broader range of non-uniform stiffness tails. Next, we determine the performance limit that can be realized by varying the chordwise stiffness of a flapping tail. To this end, we consider the performance of tails having a chordwise exponential distribution of the flexural rigidity that takes the form of $EI(x) = a e^{b x}$ where *a* is the flexural rigidity at the tail’s leading edge, *b* is the exponential rate, and *x* is the coordinate along the tail chordwise direction and varies between 0 and *c*. The limits of *a* an *b* were set to

10 $$\begin{aligned} a = [3, \ 25]\times 10^{-3} \ \ N m^2 \ \ \ \ \text {and} \ \ \ \ b = [-4.946, \ 2.026] \ \ m^{-1} \end{aligned}$$

**Fig. 6:**
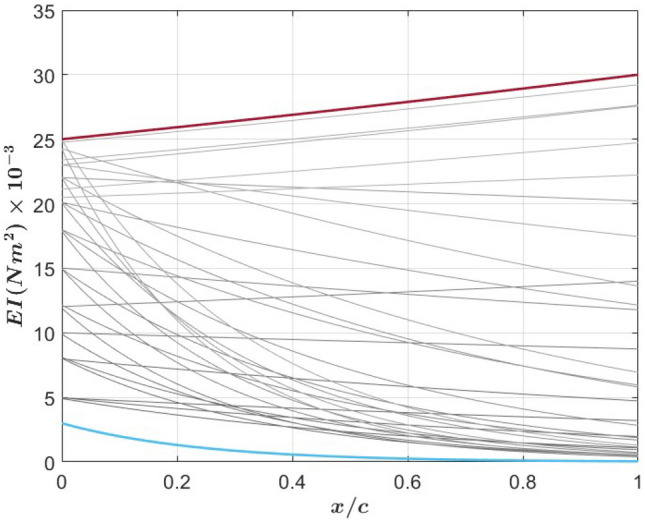
A subset of the considered varying flexural rigidity profiles showing the breadth of simulated tails.

The upper limit of the leading edge flexural rigidity, *a*, was based on initial assessment of uniform flexibility levels that lead to improved performance in terms of thrust generation or efficiency. The negative *b* values indicate a decrease in stiffness and the smaller *b* is, the smaller is the stiffness at the trailing edge. The exponential rate *b* was chosen so that the flexural rigidity at the trailing edge cannot be lower than $0.4\times 10^{-3} Nm^2$ ($\Pi _1 = 0.06$) in order to avoid a deflection that would not conform to the model assumption. The performance is assessed at three reduced frequencies, namely $k = 3.6, 4$, and 4.5 while keeping the mass ratio constant at 81. A subset of the considered rigidity profiles is presented in Fig. 6.

**Fig. 7:**
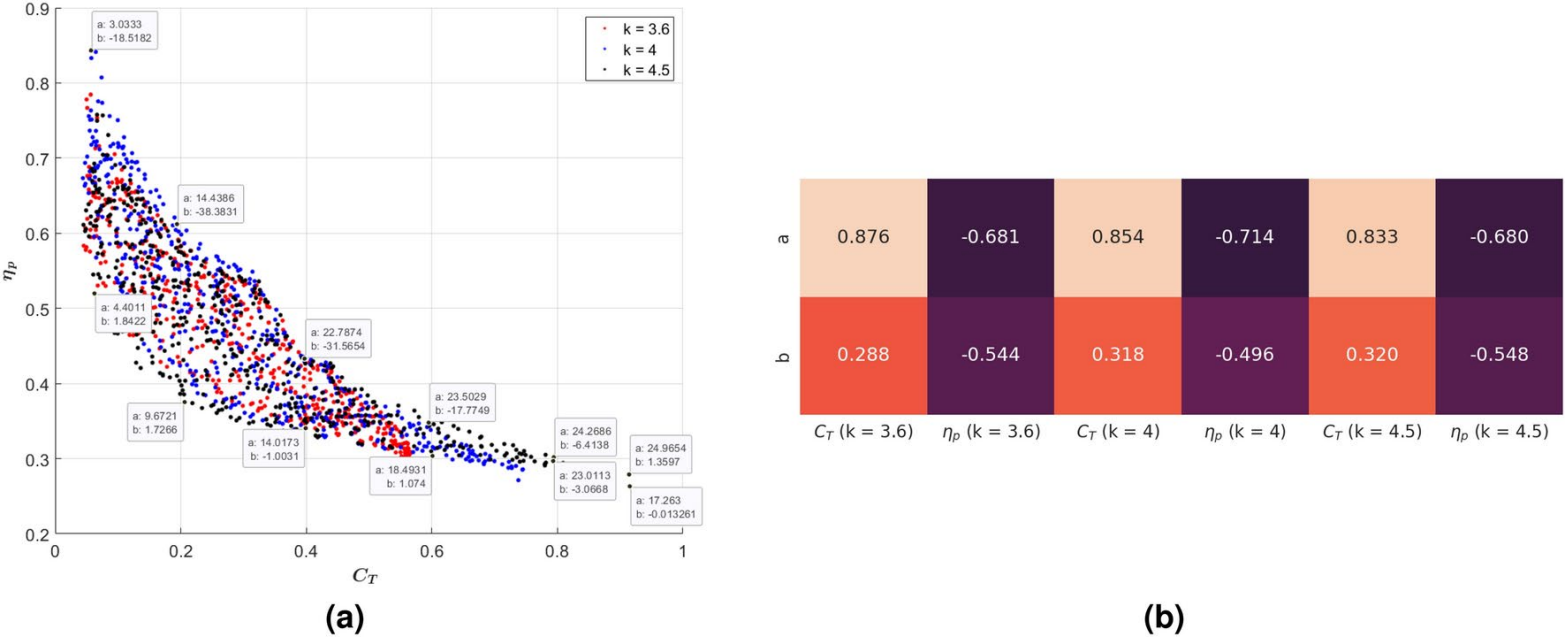
(**a**) Scatter plot of the propulsive efficiency vs thrust coefficient for over 500 profiles of chordwise varying stiffness of flexible tails excited at reduced frequencies $k = 3.6$, $k = 4$, and $k = 4.5$, and (**b**) heat map of correlation coefficients showing the impact of varying leading edge stiffness and exponential rate on thrust generation and efficiency.

**Fig. 8:**
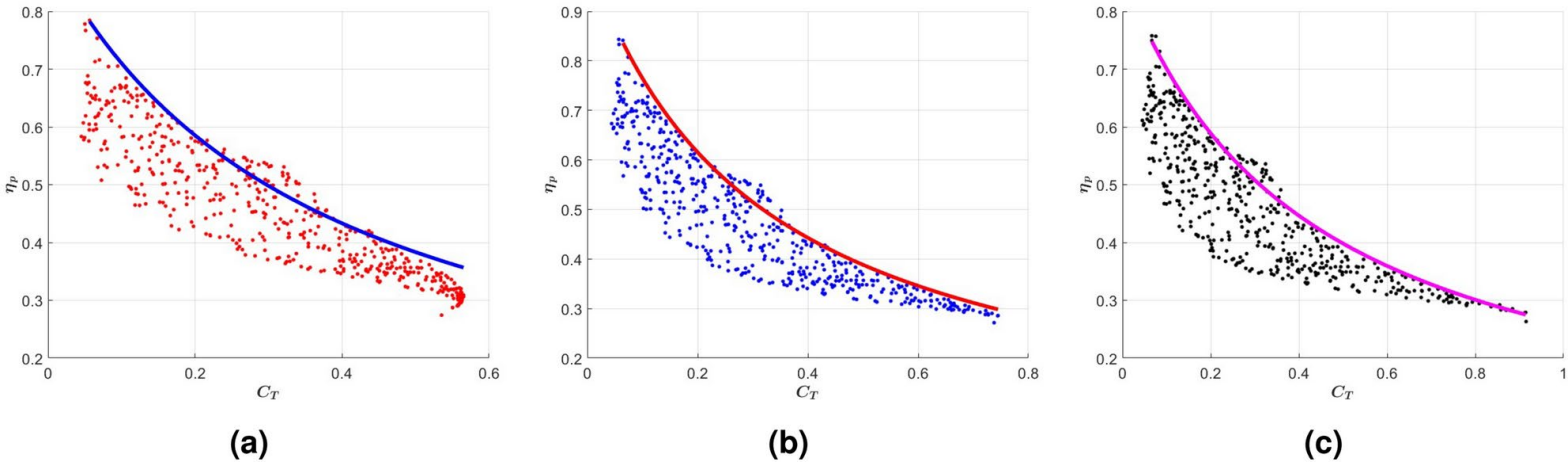
Fit of the maximum efficiency vs. thrust of considered varying stiffness profiles when excited at (**a**) $k = 3.6$, (**b**) $k = 4$, and (**c**) $k = 4.5$. Expressions for these fits are given in equation 11.

A scatter plot of the realized efficiencies versus the thrust coefficient of all nonuniform stiffness profiles considered for $k = 3.6$, $k = 4$, and $k = 4.5$ is presented in Fig. 7a. The plot underlies the significant counteraction between the thrust coefficient and efficiency in that an increase in one, beyond specific values, is in general associated with a decrease in the other. For example, generating a thrust that has a coefficient greater than 0.7 can only take place at an efficiency less than $30\%$. In addition, a high efficiency requirement such as $80\%$ can only be achieved if the thrust coefficient is below 0.1. Furthermore, generating thrust with a coefficient greater than 0.6 can only be achieved if the reduced frequency is larger than $k = 4$ at a lower efficiency level below $30\%$. Between these limits, the propulsive efficiency for a set thrust requirement varies over a relatively wide range. Similarly, the thrust coefficient for a specified efficiency value can vary over a relatively wide range. Although these variations are highly dependent on the stiffness distribution or variation, there is a maximum efficiency that can be associated with a specific thrust coefficient. The fittings of the maximum efficiency versus the thrust coefficient, superimposed on the scatter plot for the reduced frequencies $k = 3.6$, $k = 4$ and $k = 4.5$ and shown in Fig. 8, yield the relations

11 $$\begin{aligned} \begin{aligned} \eta _{max} = \frac{0.33}{C_T+0.37} \ \ \ \ \ \ \text {for} \ \ \ k = 3.6 \ \ \ \ \text {and} \ \ \ \ 0.05< C_T< 0.55\\ \eta _{max} = \frac{0.32}{C_T+0.31} \ \ \ \ \ \ \ \ \text {for} \ \ \ k = 4 \ \ \ \ \text {and} \ \ \ \ 0.05< C_T< 0.72\\ \eta _{max} = \frac{0.37}{C_T+0.43} \ \ \ \ \ \ \text {for} \ \ \ k = 4.5 \ \ \ \ \text {and} \ \ \ \ 0.05< C_T < 0.9\\ \end{aligned} \end{aligned}$$

which show the maximum efficiency for a set thrust is relatively independent of the reduced frequency over the considered range between 3.6 and 4.5.

**Fig. 9:**
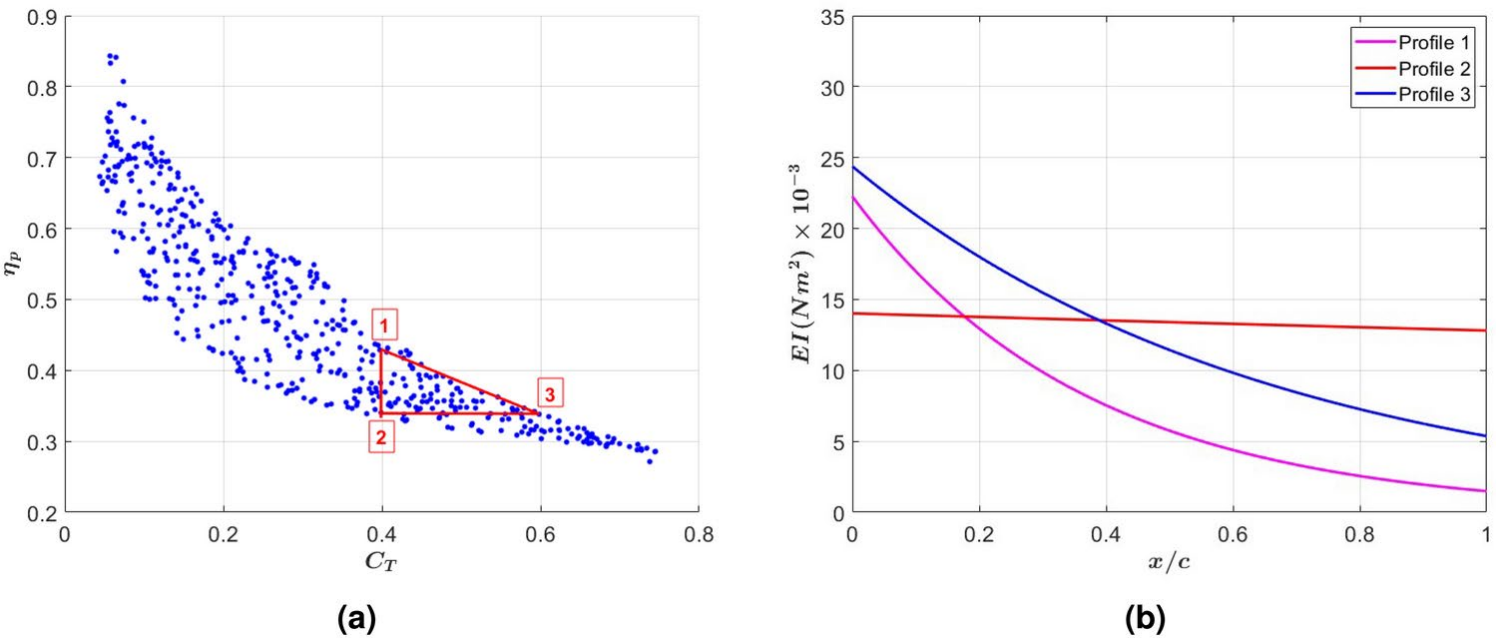
(**a**) Scatter plot of propulsive efficiency vs thrust coefficient for profiles of chordwise varying stiffness of flexible tails excited at reduced frequency $k = 4$, and (**b**) three flexural rigidity profiles corresponding to the points shown on the scatter plot. The parameters of the stiffness profiles and corresponding thrust coefficient and efficiency are presented in Table 1.

In Fig. 7b, we present a heat map showing the correlation between the thrust coefficient on the one hand, and efficiency and stiffness at the leading edge *a* and its exponential rate *b* on the other hand. This map serves to quantify the effects of varying the stiffness distribution represented by the stiffness at the leading edge *a* and its exponential rate *b*, and the observed counteraction between thrust and efficiency, as noted in the discussion of Fig. 7a. The correlation values are mostly independent of the reduced frequency. They show a significant positive correlation ($>0.8$) between the thrust generated and the rigidity or stiffness of the leading edge, which implies that increasing the stiffness of the leading edge leads to increased thrust generation. In contrast, the relatively large negative correlation ( 0.7) between propulsive efficiency and the rigidity of the leading edge shows that increasing the stiffness at the leading edge leads to reduced efficiency. The correlation values with the exponential rate *b* are smaller (between 0.25 and 0.55) than those with the stiffness of the leading edge. However, they follow the same trend in that for a defined leading edge stiffness, decreasing the stiffness at a smaller rate in the chordwise direction, i.e. a stiffer trailing edge, increases the thrust but reduces the efficiency.

This noted counteraction between the thrust coefficient and efficiency, as represented by the fitted curve and correlation trends, poses constraints on optimizing the distributed stiffness. To further elaborate, Fig. 9a shows a scatter plot of the efficiency and thrust coefficient at $k = 4$ as obtained over the range of leading edge stiffness and exponential rates defined in equation 10. In the plot, we note points 1, 2 and 3 with efficiency and thrust coefficient values respectively generated by the rigidity profiles 1, 2, and 3 presented in Fig. 9b. The parameters of the stiffness profiles and corresponding thrust coefficient and efficiency are presented in Table 1. Comparison of profiles 2 and 3 reveals that for a set efficiency level ($34\%$) the thrust can be improved by about $50\%$ by increasing the stiffness of the leading edge and reducing the stiffness of the trailing edge. Comparing profiles 3 and 1 reveals that further reduction of the trailing edge stiffness will increase efficiency but reduce thrust coefficient. Furthermore, comparing profiles 2 and 1 reveals that improving the tail’s propulsive efficiency without loosing thrust can be achieved by reducing the stiffness of the trailing edge but the maximum gain in the efficiency is limited to about $25\%$. These results show that the chordwise distribution can be varied to improve thrust or efficiency but these improvements are limited and depend on prescribed or set values of the efficiency and/or thrust coefficient.

**Table 1:** Parameters of selected stiffness profiles at $k = 4$.

	Profile 1	Profile 2	Profile 3
CT	0.4	0.4	0.6
$\eta (\%)$	43	34	34
a	22.26	14	24.38
b	− 30.08	− 1	− 16.82

**Fig. 10:**
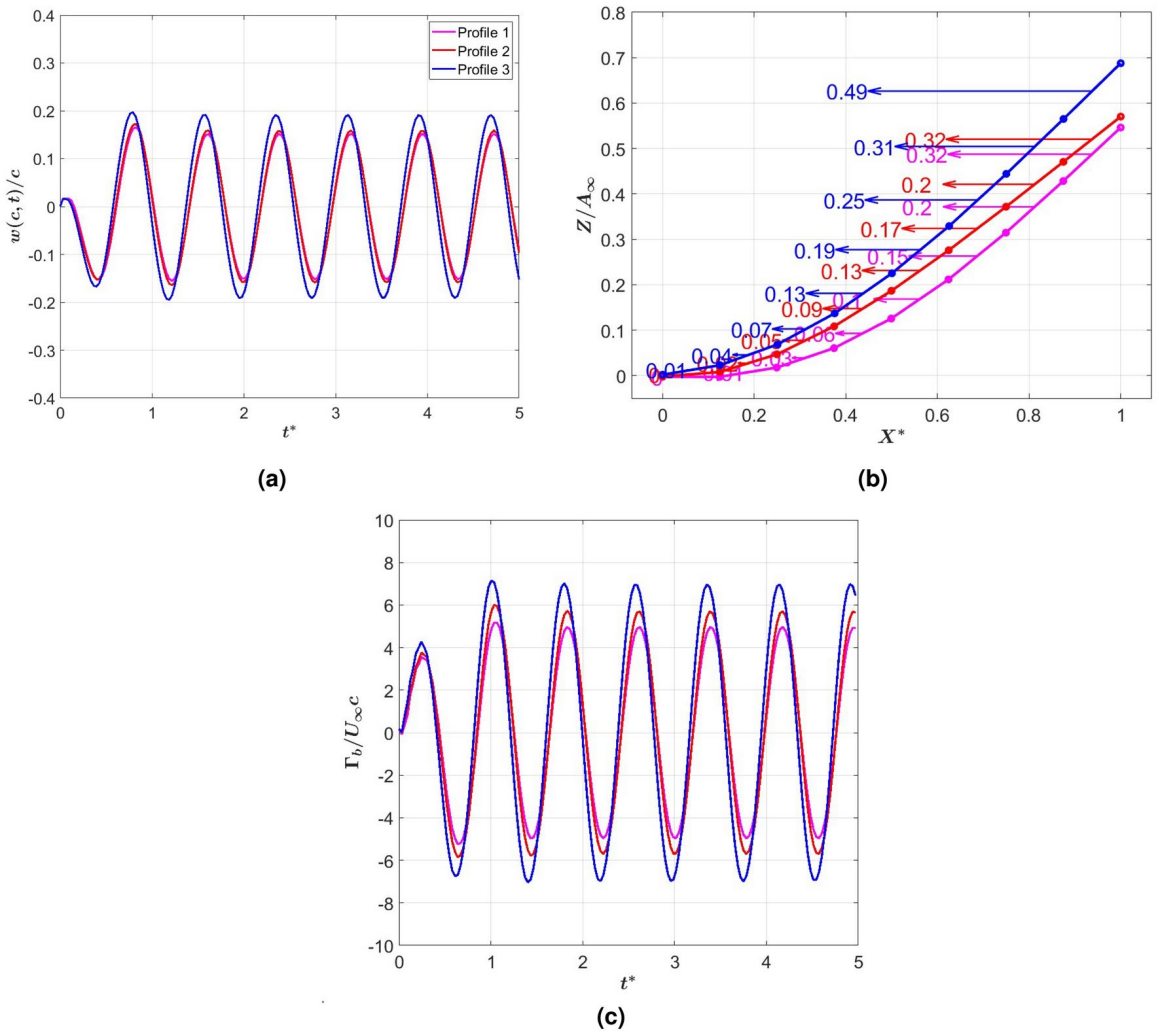
(**a**) Time history of tail’s tip deflection of the three profiles at $k = 4$, (**b**) Distribution of thrust coefficient on tail elements at maximum deformation of selected stiffness profiles, and (**c**) Time history of bound circulation at last element of the tail.

**Fig. 11:**
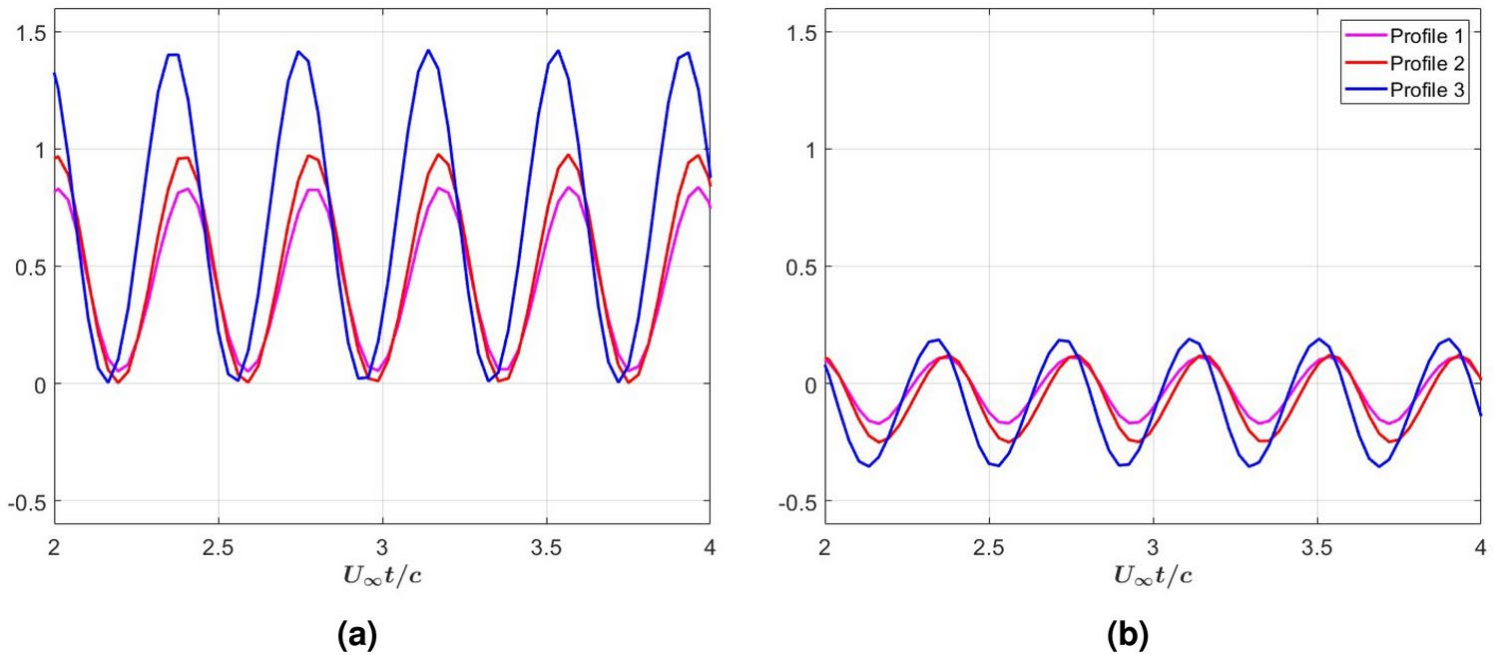
(**a**) Added mass load, and (**b**) wake load versus non-dimensional time of selected stiffness profiles at $k = 4$.

To relate the observed increased efficiency and thrust generation by varying the rigidity profile of the beam to its deflection and flow quantities, we show in Fig. 10a the time history of the trailing edge deflection for the three selected profiles. Although the tail with stiffness profile 3, undergoes the highest peak-to-peak amplitude, the differences in the amplitudes do not fully justify the difference in thrust generation. Taking into account the plots in Fig. 10a and b, it is noted that the trailing edge of the tail with stiffness profile 2 has a slightly larger peak-to-peak amplitude but a slightly lower slope than the tail with stiffness profile 1. Although both profiles lead to equal generated thrust, the increased slope and the reduced peak-to-peak amplitude increase the efficiency by about 25%, as noted in Table 1. This observation denotes that while thrust generation depends on the peak-to-peak amplitude, the efficiency can be enhanced by achieving this amplitude via stiffness variations leading to increased tail slope. Regarding the generated circulation, Fig. 10c compares the time histories of the generated bound circulation by the last tail element of the three profiles. It is noted that the circulation generated by the tail with stiffness profile 3 is the highest. However, this increased thrust requires larger power that yields the same efficiency as the tail with stiffness profile 2. When comparing profiles 1 and 2, which generate equal thrusts, it is observed that the circulation generated by profile 1 is smaller, making it a more efficient profile.

The effects of varying the stiffness in the three profiles on the added mass (non-circulatory) and wake load (circulatory) components of the forces generated at $k = 4$ are compared in the graphs of Fig. 11. The graphs in Fig. 11a show that profile 3 generates an added mass that is about 30% larger than the added mass generated by profiles 1 and 2. The graphs in Fig. 11 show that the circulatory loads of profile 3 are significantly smaller than those of the added mass. Table 2 shows the mean values of the added mass and circulatory loads generated along with their ratios. Clearly, profile 3, which generates the largest added mass, has a high ratio of non-circulatory to circulatory loads. Among the three profiles, profile 3 generates the largest thrust (Table 1). Based on these observations, it is concluded that the improvement in thrust is mostly achieved by the increased added mass load without a large contribution from the circulatory loads. Because profile 3 has a lower efficiency than profile 1 (Table 1) and a lower ratio of added mass to circulatory loads (Table 2), it is concluded that improving efficiency is associated with decreasing the ratio (in the absolute sense) of the wake load (circulatory) to the added mass load (non-circulatory).

**Table 2:** Mean values of added mass and wake load for selected stiffness profiles at $k = 4$.

	Profile 1	Profile 2	Profile 3
Added mass (noncirculatory)	0.43	0.47	0.68
Wake (circulatory)	− 0.03	− 0.07	− 0.09
Ratio (absolute)	0.07	0.15	0.13

## Conclusions

We performed numerical simulations to assess the effects of the chordwise stiffness distribution on the hydrodynamic performance of a flapping fish tail. The tail was modeled as a deforming beam subjected to pitching excitation at its root while moving forward at a constant speed. The results show that increased thrust in the case of uniform stiffness tails correlates well with increased peak-to-peak amplitude in the range of conducted simulations covering reduced frequency up to $k = 4.5$, which corresponds to a Strouhal number of about 0.4. In addition, the required flapping power to generate thrust correlates well with generated circulation. Non-uniform stiffness tails, with decreased stiffness in the chordwise direction, exhibit higher efficiency levels mostly because they generate higher thrust even when the peak-to-peak amplitude or wake circulation are reduced. This ability is due to their passive deflections that give the trailing edge a favorable high slope to support thrust generation. Based on correlation heat maps from hundreds of simulations, it was demonstrated that increasing the stiffness at the leading edge enhances the thrust but decreases the efficiency. In contrast, decreasing the stiffness in the chordwise direction at a higher rate along the tail length increases the propulsion efficiency, but decreases the thrust. The enhanced performance is associated with an increased contribution by added mass effects relative to the circulatory contribution. In particular, while increased added mass is associated with increased thrust, it is associated with decreased efficiency. The extensive simulations were used to derive expressions for this counteractive relation in terms of maximum attainable efficiency for a set thrust coefficient. The analysis leading to such expressions can be used to assess the performance of different configurations in the initial stages of the design of oscillatory bioinspired swimming robots. Furthermore, the approach and presented analysis can be used in the optimization of geometry and material properties of chordwise-varying stiffness tail to yield maximum efficiency under specific thrust constraint.

## Data Availability

The data that support the findings of this study are available from the corresponding author, H.A., upon reasonable request.
